# Bone morphogenetic protein-2-induced Wnt/β-catenin signaling pathway activation through enhanced low-density-lipoprotein receptor-related protein 5 catabolic activity contributes to hypertrophy in osteoarthritic chondrocytes

**DOI:** 10.1186/ar3805

**Published:** 2012-04-18

**Authors:** Ioanna Papathanasiou, Konstantinos N Malizos, Aspasia Tsezou

**Affiliations:** 1 Laboratory of Cytogenetics and Molecular Genetics, University of Thessaly, School of Medicine, Mezourlo, Larissa, 41100, Greece; 2 Department of Orthopaedics, University of Thessaly, School of Medicine, Mezourlo, Larissa, 41100, Greece; 3 Institute for Biomedical Research and Technology, Papanastasiou 51, Larissa, 41222, Greece; 4 Department of Biology, University of Thessaly, School of Medicine, Mezourlo, Larissa, 41100, Greece

## Abstract

**Introduction:**

Events normally taking place in the terminal chondrocyte differentiation in the growth plate are also observed during osteoarthritis (OA) development, suggesting that molecules, such as Wnts and bone morphogenetic proteins (BMPs) regulating chondrocyte activity in the growth plate, may play a key role in osteoarthritis pathogenesis. The aim of the study was to investigate the possible cross-talk between BMP-2 and Wnt/β-catenin pathways in OA progression.

**Methods:**

Low-density-lipoprotein receptor-related protein 5 (LRP-5) and 6, BMP-2, -4, and -7, bone morphogenetic protein receptor-IA and IB (BMPR-IA and BMPR-IA), lymphoid enhancer factor-1 (LEF-1), and transcription factor 4 **(**TCF-4) expression levels were investigated in normal and osteoarthritic chondrocytes. LRP-5, β-catenin (phospho and active form), matrix metalloproteinases (MMPs) 7, 9, 13, 14, ADAMTS-4, 5, as well as collagen X (COL10A1) expression levels were evaluated after LRP-5 silencing in BMP-2-treated chondrocytes. The investigation of Smad1/5/8 binding to *LRP-5 *promoter was assessed with chromatin immunoprecipitation (ChIP). Furthermore, we evaluated the effect of experimental activation of the Wnt/β-catenin pathway with LiCl and LEF-1 silencing, in LiCl-treated chondrocytes, on matrix metalloproteinases (MMPs) 7, 9, 13, 14, ADAMTS-4, 5, and collagen X (COL10A1) expression, as well as possible interactions between LEF-1 and *MMPs *and *COL10A1 *promoters by using a ChIP assay.

**Results:**

LRP-5, BMP-2, BMP-4, BMPR-IA, and LEF-1 mRNA and protein expression levels were found to be significantly upregulated in osteoarthritic chondrocytes compared with normal. We showed that treatment of cultured chondrocytes with BMP-2 resulted in increased β-catenin nuclear translocation and LRP-5 expression and that the BMP-2-induced LRP-5 upregulation is mediated through Smad1/5/8 binding on *LRP-5 *promoter. LRP-5 silencing reduced nuclear β-catenin protein levels, MMPs and collagen X expression, whereas increased phospho-β-catenin protein levels in BMP-2-treated chondrocyte. Furthermore, we demonstrated that activation of the Wnt/β-catenin signaling pathway by LiCl and LEF-1 downregulation by using siRNA regulates MMP-9, 13, 14, ADAMTS-5, and COL10A1 expression, evidenced by the observed strong binding of LEF-1 to *MMP-9, 13, 14, ADAMTS-5 *and *COL10A *promoters.

**Conclusions:**

Our findings suggest, for the first time to our knowledge, that BMP-2-induced Wnt/β-catenin signaling activation through LRP-5 may contribute to chondrocyte hypertrophy and cartilage degradation in osteoarthritis.

## Introduction

Osteoarthritis (OA) is a progressively degenerative joint disorder characterized by extracellular matrix degradation, articular cartilage loss, and osteophyte formation [1]. It is considered a major health problem worldwide, causing chronic disability in elderly people [2]. However, the molecular mechanisms underlying OA pathogenesis are poorly understood, and no disease-modifying therapy is currently available [1,2].

Osteoarthritis involves mainly the dysfunction of articular chondrocytes, which leads to cartilage degradation through chondrocyte maturation and MMPs production [3,4]. In growth-plate chondrogenesis, chondrocytes become hypertrophic, expressing collagen X, remove the collagen matrix through the production of MMP-13, and finally die by apoptosis and are replaced by bone [5,6]. Alternatively, chondrocytes of permanent cartilage reside at the ends of the long bones and do not mature into the hypertrophic state, preventing terminal differentiation by an unknown mechanism [7]. However, during osteoarthritis, chondrocytes lose the stable phenotype and undergo changes that occur in terminal differentiated growth-plate chondrocytes, such as high expression of MMP-13 and collagen X [8-10].

The function of articular chondrocytes is regulated by different growth factors, including Wnt ligands and BMPs, which have been shown to play a critical role in chondrocyte proliferation, differentiation, and apoptosis [11-13]. The canonic or Wnt/β-catenin pathway signals through Frizzled family receptors and coreceptors LRP-5/LRP-6 and leads to stabilization of β-catenin, which in turn interacts with transcription factors, such as LEF-1/TCF-4 proteins, and activates specific genes, as *c-myc *and *cyclin D1 *[14]. The role οf Wnt/β-catenin signaling pathway in OA development has been previously suggested, as an association has been reported between hip OA susceptibility in women and two functional genetic variants in *FRZB*, which encode frizzled-related protein, a soluble antagonist of the Wnt canonic signaling pathway [15-17]. Additional evidence for the involvement of Wnt/β-catenin signaling in OA comes from the observation that *frzd *knockout mice are more susceptible to chemically induced OA [18]. Besides FRZB, another antagonist of the pathway, dickkopf-related protein 1 (Dkk-1), has been also shown to be associated with reduced progression of OA in elderly women when it is present in elevated levels in the serum [19]. A recent study showed the implication of SOST, a potent inhibitor of canonic Wnt signaling by binding to LRP5/6 in OA disease processes with opposing effects by promoting disease-associated subchondral bone sclerosis and inhibiting degradation of cartilage [20]. Recently, we showed that coreceptor of the Wnt/β-catenin signaling pathway, LRP-5, may have a catabolic role in osteoarthritis, as we observed significant upregulation of LRP-5 expression in osteoarthritic chondrocytes [21]. Moreover, the involvement of the Wnt/β-catenin signaling pathway in the regulation of cartilage development and homeostasis has been confirmed, as increased expression levels of several Wnt proteins and Frizzled receptors have been found in the synovial tissue of arthritic cartilage [22], and the conditional activation of the β*-catenin *gene in articular chondrocytes in adult mice leads to premature chondrocytes differentiation and the development of an OA-like phenotype [23]. Increased levels of β-catenin have been reported in chondrocytes within areas of degenerative cartilage, and its accumulation and transcriptional activity has been shown to stimulate chondrocyte matrix catabolic action, to induce the expression of different MMPs in articular chondrocytes, and to promote hypertrophic differentiation of chondrocytes, evidenced by increased expression of collagen X [24-26].

Besides the Wnt/β-catenin pathway, BMPs also play a significant role in chondrocyte differentiation. BMP signaling is initiated by BMPs binding to their receptor (BMPR-I and II), resulting in increased phosphorylation of downstream signaling molecules, including Smad1, Smad5, and Smad8 (R-Smads). The R-Smads form complexes with Smad-4 and translocate into the nucleus, where they bind to regulatory regions of target genes regulating their expression [27]. Although BMPs are considered to have a protective effect in articular cartilage, it has been proposed that they are also involved in chondrocyte hypertrophy and matrix degradation [28-31]. Chen *et al. *[32] reported BMP-2, 4-6, and 11 mRNA expression in normal and osteoarthritic adult human cartilage, whereas Steinert *et al. *[33] showed that BMP-2 and BMP-4 induce hypertrophy during the *in vitro *chondrogenic differentiation of human mesenchymal stem cells. Moreover, in preosteoblastic cells, BMP-2 was found to increase nuclear β-catenin protein levels and induce the expression of different Wnts, suggesting the interaction between Wnt and BMP-2 signaling during osteoblastic differentiation [34].

As activation of Wnt/β-catenin and BMP-2 signaling pathways may signify a causal relation to chondrocyte differentiation and matrix degradation, we sought to investigate the possible cross-talk between BMP-2 and Wnt/β-catenin pathways in the catabolic action and hypertrophy of osteoarthritic chondrocytes.

## Materials and methods

### Bioinformatic analysis

Promoter sequences of *MMP-7, 9, 13, 14, ADAMTS-5, 4*, and *COL10A1 *genes were obtained from CHIP Bioinformatics Tools [35]. For each of these genes, we scanned the region from 1,500 base pairs upstream of the transcript start to 100 base pairs downstream of the coding-sequence start to find putative LEF-1 binding elements (5'-CTTTGWW-3'). In addition, *LRP-5 *promoter was tested for Smad binding sites (SBEs, 5'-GCCGnGCG-3').

### Patients and cartilage samples

Articular cartilage samples were obtained from femoral condyles and tibial plateaus of patients with primary osteoarthritis undergoing knee-replacement surgery at the Orthopaedics Department of University Hospital of Larissa. In total, 18 patients were included in this study (15 women/three men; mean age, 64.7 ± 9.7 years). All osteoarthritic specimens had Mankin scores of 10 to 14. Radiographs were obtained before surgery and graded according to the Kellgren-Lawrence system. All patients had K/L scores ≥ 2. The radiographs were assessed by two independent observers who were blinded to all data of the individuals. Normal articular cartilage was obtained from nine individuals (four women/five men; mean age, 44.6 ± 7.6 years) with 0 Mankin score, undergoing fracture-repair surgery, with no history of joint disease. Patients with rheumatoid arthritis and other autoimmune diseases, as well as chondrodysplasias, infection-induced OA, and posttraumatic OA, were excluded from the study. Written informed consent was obtained from all individuals in the study. The study protocol conformed to the ethical guidelines of the 1975 Declaration of Helsinki, as reflected in *a priori *approval by the Local Ethical Committee of the University Hospital of Larissa.

### Primary cultures of normal and osteoarthritic human articular chondrocytes

Articular cartilage was dissected and subjected to sequential digestion with 1 mg/ml proteinase mixture (Pronase; Roche Applied Science, Mannheim, Germany) and 1 mg/ml collagenase P (Roche Applied Science, Mannheim, Germany). Chondrocytes were counted and checked for viability by using trypan blue staining. More than 95% of the cells were viable after isolation. Isolated chondrocytes from individual specimens were separately cultured with Dulbecco Modified Eagle Medium/Ham F-12 (DMEM/F-12) (GIBCO BRL, Paisley, UK) plus 5% fetal bovine serum (FBS; Invitrogen, Life Technologies, Paisley, UK) at 37^ο^C under a humidified 5% CO_2 _atmosphere until reaching confluence for 4 to 6 days.

### RNA extraction and quantification of mRNA expression

Total cellular RNA was extracted from cultured chondrocytes by using Trizol reagent (Invitrogen, Life Technologies, Paisley, UK). Preservation of 28S and 18S ribosomal RNA (rRNA) species was used to assess RNA integrity. All the samples included the study had prominent 28S and 18S rRNA components. The yield was quantified spectrophotometrically. Transcription of 0.1 μg RNA to cDNA was performed by using SuperScript III reverse transcriptase (Invitrogen, Life Technologies, Paisley, UK) and random primers (Invitrogen, Life Technologies, Paisley, UK). Quantification of COL2A1, COL10A1, aggrecan, MMP-13, LRP-5, LRP-6, BMP-2, BMP-4, BMP-7, BMPR-IA, BMPR-IB, LEF-1, and TCF-4 mRNA expression was performed with real-time PCR (ABI 7300; Applied Biosystems, Foster City, CA, USA). Reactions were done in triplicate by using 2 μl of cDNA per reaction. All primers used are shown in Table 1. Real-time PCR validation was carried out by using the 2^-ΔΔCT ^method. Normalized gene-expression values for each gene based on cycle threshold (C_T_) values for each of the genes and the housekeeping gene glyceraldehyde 3-phosphate dehydrogenase (GAPDH) were generated.

**Table 1:** Oligonucleotide primers used in real-time PCR assay

Gene	Forward primer sequence	Reverse primer sequence
***BMP-2***	CCCAGCGTGAAAAGAGAGAC	GGAAGCAGCAACGCTAGAAG

***BMPR-IA***	TTTATGGCACCCAAGGAAAG	TGGTATTCAAGGGCACATCA

***LEF-1***	CCCTTCCAACTCTCCTTTCC	TTGAAGGGGATCATCTCGTC

***Osteocalcin***	AGAGTCCAGCAAAGGTGCAG	TCAGCCAACTCGTCACAGTC

***LRP-5***	GTGCCTGGGTGAGATTCTTC	CACGAAGACTGCGAAACAGA

***MMP-7***	TGCTCACTTCGATGAGGATG	TGGGGATCTCCATTTCCATA

***MMP-13***	TGGCATTGCTGACATCATGA	GCCAGAGGGCCCATCAA

***MMP-14***	GAGCTCAGGGCAGTGGATAG	GGTAGCCCGGTTCTACCTTC

***MMP-9***	TTGACAGCGACAAGAAGTGG	GCCATTCACGTCGTCCTTAT

***BMP-4***	ATGAAGCCCCCAGCAGAAGT	TCACATTGTGGTGGACCAGTC

***ADAMTS-4***	TCCTGCAACACTGAGGACTG	GGTGAGTTTGCACTGGTCCT

***ADAMTS-5***	CAGCAGTGCAACCTGACATT	CAGATTCTCCCCTTTCCACA

***COL10A1***	CAGGCATAAAAGGCCCAC	GTGGACCAGGAGTACCTTGC

***COL2A1***	ATGACAATCTGGCTCCCAACACTGC	GACCGGCCCTATGTCCACACCGAAT

***Aggrecan***	TGAGGAGGGCTGGAACAAGTACC	GGAGGTGGTAATTGCAGGGAACA

***GAPDH***	GAGTCAACGGATTTGGTCGT	GACAAGCTTCCCGTTCTCAG

### Protein extraction and Western blot analysis

Chondrocytes were lysed by using RIPA buffer and a cocktail of protease and phosphatase inhibitors. Protein concentration was quantified by using the Bio-Rad Bradford protein assay (Bio-Rad Protein Assay; BioRad, Hercules, CA, USA) with bovine serum albumin as standard. Cell lysates from normal and OA chondrocytes were electrophoresed and separated on a 4% to 20% Tris-HCl gel (Bio-Rad, Hercules, CA, USA) and transferred to a Hybond-ECL nitrocellulose membrane (Amersan Biosciences, Piscataway, NJ, USA). The membrane was probed with anti-LRP-5 (sc-21389; Santa Cruz Biotechnology, Santa Cruz, CA, USA), BMP-2 (sc-57040; Santa Cruz Biotechnology, Santa Cruz, CA, USA), BMP-4 (AF757, R&D Systems, Minneapolis, MN, USA) BMPR-IA (sc-134285; Santa Cruz Biotechnology, Santa Cruz, CA, USA), LEF-1 antibody (sc-8591; Santa Cruz Biotechnology, Santa Cruz, CA, USA), and phospho-β-catenin (9561; Cell Signaling Technology, Boston, MA, USA), and signals were detected by using immunoglobulin (IgG) conjugated with horseradish peroxidase (1:10,000 dilution). The results were normalized by using anti-β-actin polyclonal antibody (Sigma-Aldrich, St. Louis, MO, USA). The nitrocellulose membranes were then exposed to photographic film, which was scanned, and the intensities of the protein bands were determined with computerized densitometry.

### Immunohistochemistry

Cartilage specimens were fixed in 10% formalin overnight and decalcified in 13% EDTA (Sigma-Aldrich, St. Louis, MO, USA) for 3 weeks. Paraffin-embedded sections were deparaffinized in xylene, dehydrated through graded alcohols, and placed in 3% H_2_O_2 _to block endogenous peroxidase. Nonspecific staining was blocked with TBS containing 2% bovine serum albumin (BSA; Sigma-Aldrich, St. Louis, MO, USA) for 1 hour at room temperature, followed by incubation with primary antibody BMP-2 (c-57040; Santa Cruz Biotechnology, Santa Cruz, CA, USA; 1:20 dilution with TBS containing 2% BSA) overnight at room temperature. Normal serum of the same species as the primary antibody was used as a control for the primary antibody. After extensive washing with TBS, the sections were incubated with horseradish peroxidase-conjugated secondary antibody (Goat anti-Mouse; Invitrogen, Life Technologies, Paisley, UK; 1:250 dilution with TBS containing 2% BSA) for 1 hour at room temperature in a humid chamber. Finally, color reaction was performed by using the substrate reagent 3,3'-diaminobenzidine tetrahydrochloride (DAB; Thermo Scientific, Rockford, IL, USA). After being washed, the sections were incubated with DAB and coverslipped with mounting medium.

### Chondrocyte treatment with BMPs

Normal and osteoarthritic chondrocytes were seeded on six-well plates at 3 × 10^5 ^cells/well, and 3 days after seeding, cells were treated with 50 ng/ml of BMP-2 or BMP-4 for 12, 24, and 48 hours; each experiment was conducted in triplicate wells. RNA was extracted, and real-time PCR analysis for osteocalcin, LRP-5, and LRP-6 was performed, as described earlier. All primers used are shown in Table 1. Cell lysates were extracted, and LRP-5 (sc-21389; Santa Cruz Biotechnology, Santa Cruz, CA, USA), phospho-β-catenin (9561; Cell Signaling Technology, Boston, MA, USA), and total β-catenin protein levels (9582; Cell Signaling Technology, Boston, MA, USA) were evaluated by using Western blot analysis, as described earlier.

### Chondrocyte treatment with LiCl

Normal and osteoarthritic chondrocytes were seeded on six-well plates at 3 × 10^5 ^cells/well, and 3 days after seeding, cells were treated with 20 m*M *LiCl (Sigma-Aldrich, St. Louis, MO, USA) for 12, 24, and 48 hours; each experiment was conducted in triplicate wells. RNA was extracted, and real-time PCR analysis for MMP-7, 9, 13, 14, ADAMTS-4, 5, and collagen X was performed as described earlier. All primers used are shown in Table 1. Cell lysates were extracted, and phospho-β-catenin protein levels were evaluated by using Western blot analysis, as described earlier.

### Chromatin immunoprecipitation (ChIP) assay

ChIP was performed by using a ChIP assay kit (Upstate USA, Inc., Charlottesville, VA, USA) on BMP-2-treated, LiCl-treated, and untreated normal chondrocytes, as previously described [21]. The cell lysates after treatment with BMP-2 or LiCl were precleared by incubation with G-Sepharose beads and were incubated with monoclonal antibody Smad-1/5/8 (Cell signaling Technology, Boston, MA, USA) or polyclonal antibody LEF-1 overnight at 4°C, respectively. Antibody to human purified IgG was used as control (R&D Systems, Minneapolis, MN, USA). The immunoprecipitated DNAs were used for PCR amplification. All primers were designed according to the nucleotide sequence of the gene promoters, and each PCR fragment covered 250 to 400 bp of the promoter. Table 2 shows only the primer sets that amplify the promoter region containing putative sites, as observed after ChIP assay. The PCR products were fractionated on 3% agarose gels and were stained with ethidium bromide.

**Table 2:** Oligonucleotide primers used for ChIP assay

Gene	Forward primer	Reverse primer
*MMP-13*	GGACGGTGGTCAAGAACATT	GGCTAGCACAAACAGGGATT

*MMP-9*	TAAGACATTTGCCCGAGGTC	CTCCCTGACAGCCTTCTTTG

*MMP-14*	TCTCCCACACTTTTCCTGCT	AAGAAGGGATTGGGAGTTGG

*ADAMTS-5*	TTTCTTCCCTTCCTCCTGGT	TCAGCAAATACGGGAAAAGG

*COL10A1*	GGAATTGTCCTCCTCAACCA	CACTTTCCCTCAAAGGTGGA

*LRP-5*	GTTCCAGAGCCCCTTCTGT	CCGGACACTTGTTCTCACCT

### Oligonucleotide transfections

Normal and osteoarthritic chondrocytes were seeded in six-well plates in DMEM/F-12 containing 5% FBS. After overnight incubation, cells were transfected with specific siRNA against LRP-5 (Ambion, Life Technologies, Paisley, UK), LEF-1 (Invitrogen, Life Technologies, Paisley, UK), and scrambled siRNA (used as a control; Invitrogen, Life Technologies, Paisley, UK) by using LipofectAMINE 2000 (Invitrogen, Life Technologies, Paisley, UK), as described by the manufacturer. No cell toxicity was detected from the transfection agent. Four hours after siRNA transfection, the medium was changed, and cells were treated with 50 ng/ml BMP-2 (cultures with siRNA against LRP-5) or 20 m*M *LiCl (cultures with siRNA against LEF-1) for 48 hours. In each experiment, the results from three of the six-well plates were averaged and considered as *n *= 1. No significant variance was observed among the individual wells in each averaged group. RNA was extracted, and real-time PCR analysis for MMP-7, 9, 13, 14, ADAMTS-4, 5, and collagen X was performed, as described earlier. Moreover, cell lysates were extracted, and phospho- as well as total β-catenin protein levels were evaluated in BMP-2-treated chondrocytes after LRP-5 siRNA transfection by using Western blot analysis, as described earlier.

### Statistical analysis

Statistical significance was determined by using the Student *t *test with a confidence level of 95% (*P *< 0.05).

## Results

### COL2A1, COL10A1, aggrecan, and MMP-13 expression in normal and osteoarthritic chondrocytes

**Figure 1:**
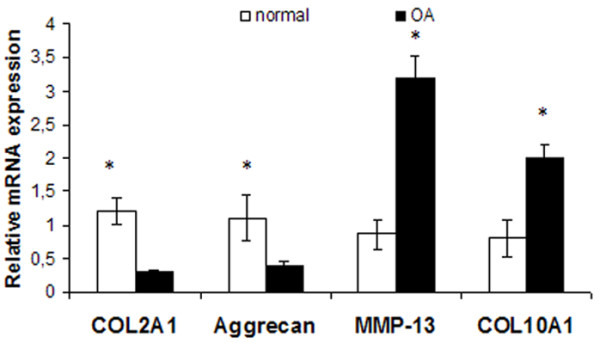
**COL2A1, COL10A1, aggrecan, and matrix metalloproteinase (MMP)-13 expression in normal and osteoarthritic chondrocytes**. Quantitative COL2A1, COL10A1, aggrecan, and MMP-13 expression in normal and osteoarthritic chondrocytes. The results showed that COL2A1 and aggrecan were significantly upregulated in normal chondrocytes, whereas COL10A1 and MMP-13 were significantly upregulated in osteoarthritic chondrocytes. (Error bars, SEM; *P < 0.05).

To evaluate differences in chondrocyte differentiation-related genes, we evaluated COL2A1, COL10A1, aggrecan, and MMP-13 mRNA expression levels in normal and osteoarthritic chondrocytes. We found that COL2A1 and aggrecan were significantly upregulated in normal chondrocytes (*P *< 0.05; Figure 1), whereas COL10A1 and MMP-13 were significantly upregulated in osteoarthritic chondrocytes (*P *< 0.05; Figure 1).

### LRP-5, BMP-2, BMP-4, BMP-7, BMPR-IA, BMPR-IB, LRP-6, phospho-β-catenin, LEF-1, and TCF-4 expression in normal and osteoarthritic chondrocytes

**Figure 2:**
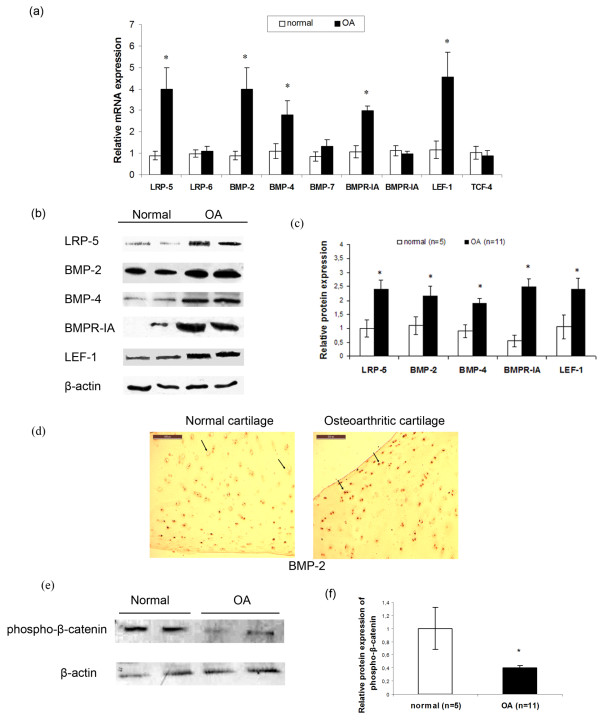
**Low-density-lipoprotein receptor-related protein (LRP)-5, BMP-2, BMP-4, BMP-7, BMPR-IA, BMPR-IB, LRP-6, phospho-β-catenin, LEF-1, and TCF-4 expression in normal and osteoarthritic chondrocytes**. **(a) **Quantitative LRP-5, BMP-2, BMP-4, BMP-7, BMPR-IA, BMPR-IB, LRP-6, LEF-1, and TCF-4 expression in normal and osteoarthritic chondrocytes. The results showed that LRP-5 (fourfold), BMP-2 (fourfold), BMP-4 (threefold), BMPR-IA (twofold), and LEF-1 (4.5-fold) mRNA expression was significantly increased in osteoarthritic chondrocytes compared with normal. (Error bars, SEM; standard errors, *P < 0.05). **(b) **LRP-5, BMP-2, BMP-4, BMPR-IA, and LEF-1 protein expression in normal and osteoarthritic chondrocytes after Western blot analysis. The results showed that LRP-5, BMP-2, BMP-4, BMPR-IA, and LEF-1 protein expression was significantly increased in osteoarthritic chondrocytes compared with normal. β-actin was used as internal control. **(c) **Bar graph showing the LRP-5, BMP-2, BMP-4, BMPR-IA, and LEF-1 protein levels in normal (*n *= 5) and osteoarthritic chondrocytes (*n *= 11). (Error bars, SEM; standard errors, *P < 0.05). **(d) **Expression of BMP-2 in normal and osteoarthritic cartilage after immunohistochemistry experiments. **(e) **Western blot analysis showed a significant reduction of phospho-β-catenin protein levels in osteoarthritic chondrocytes compared with normal. **(f) **Bar graph showing the phospho-β-catenin protein levels in normal (*n *= 5) and osteoarthritic chondrocytes (*n *= 11). (Error bars, SEM; *P < 0.05).

To investigate the involvement of BMP-2 and Wnt/β-catenin signaling pathway in osteoarthritis, we evaluated BMP-2, BMP-4, BMP-7, BMPR-IA, BMPR-IB, LRP-5, LRP-6, phospho-β-catenin, LEF-1, and TCF-4, the major transcription factors of the Wnt canonic signaling pathway expression levels in normal and osteoarthritic chondrocytes. We found that osteoarthritic chondrocytes had significantly higher LRP-5, BMP-2, BMP-4, BMPR-IA, LEF-1 mRNA, and protein expression compared with normal (*P *< 0.05; Figure 2a, b, c). Moreover, BMP-2 was found to be upregulated in osteoarthritic compared with normal cartilage after immunohistochemistry (Figure 2d). Western blot analysis showed a significant reduction of phospho-β-catenin protein levels in osteoarthritic chondrocytes compared with normal (*P *< 0.05; Figure 1e and 1f). No significant difference was found in LRP-6, BMP-7, BMPR-IB, and TCF-4 mRNA levels between normal and osteoarthritic chondrocytes (Figure 2a).

### BMP-2 modulates Wnt/β-catenin signaling pathway through stimulation of LRP-5 mRNA and protein expression in chondrocytes

**Figure 3:**
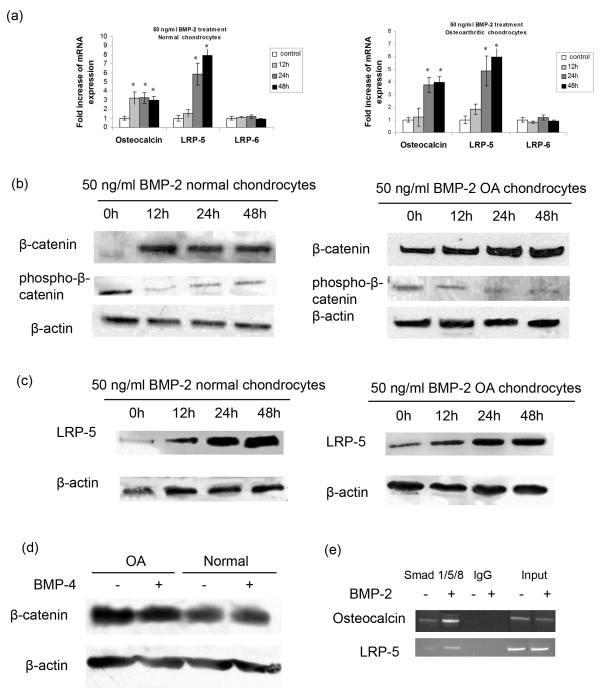
**Effect of BMP-2 treatment on β-catenin and LRP-5 expression**. **(a) **Real-time PCR of LRP-5 and LRP-6 mRNA levels in the presence and absence of BMP-2 (50 ng/ml) for 12, 24, and 48 hours in normal and osteoarthritic chondrocytes. Osteocalcin expression used as positive control. (Error bars, SEM; *P < 0.05 versus control). **(b) **and **(c) **Representative Western blots of β-catenin, phospho-β-catenin, and LRP-5 protein levels after treatment with 50 ng/ml BMP-2 for 12, 24, and 48 hours in normal and osteoarthritic chondrocytes. β-Actin was used as internal control. **(d) **ChIP assay was performed by using anti-Smad1/5/8 antibody or irrelevant anti-immunoglobin G antibody as negative control in untreated and BMP-2-treated chondrocytes for 48 hours. Input samples are total genomic DNAs used as control for the PCR. ChIP assay shows binding of transcription factor Smad1/5/8 to the *LRP-5 *promoter in BMP-2-treated chondrocytes. *Osteocalcin *promoter used as positive control.

BMP-2 and Wnt/β-catenin signaling pathways have been reported to play a significant role in the control of chondrocyte differentiation in the growth plate. We examined the effect of BMP-2 on Wnt/β-catenin signaling-pathway activation after treatment with BMP-2 in normal and osteoarthritic chondrocytes. We found that BMP-2 treatment at 12, 24, and 48 hours increased the active form of nuclear β-catenin protein levels (Figure 3b), decreased phospho-β-catenin protein levels (Figure 3b), and significantly upregulated LRP-5 mRNA and protein expression, but not LRP-6 (*P *< 0.05; Figure 3a and 3c). However, no difference was noted in the active form of nuclear β-catenin protein levels after treatment with BMP-4 (Figure 3d). Further to investigate the intracellular signaling pathway involved in BMP-2-induced LRP-5 expression, we subjected BMP-2-treated chondrocytes to chromatin immunoprecipitation by using an antibody against Smad-1/5/8 and tested whether Smads bind to *LRP-5 *promoter via Smad-binding elements and subsequently LRP-5 expression. We observed that *LRP-5 *promoter contains conserved a Smad-binding site in -280 to -270 from the ATG initiation codon, and Smad1/5/8 binding was enhanced after treatment with BMP-2. Smads binding to *osteocalcin *promoter served as positive control (Figure 3e).

### LRP-5 upregulation by BMP-2 contributed to catabolic activity and hypertrophy of osteoarthritic chondrocytes

**Figure 4:**
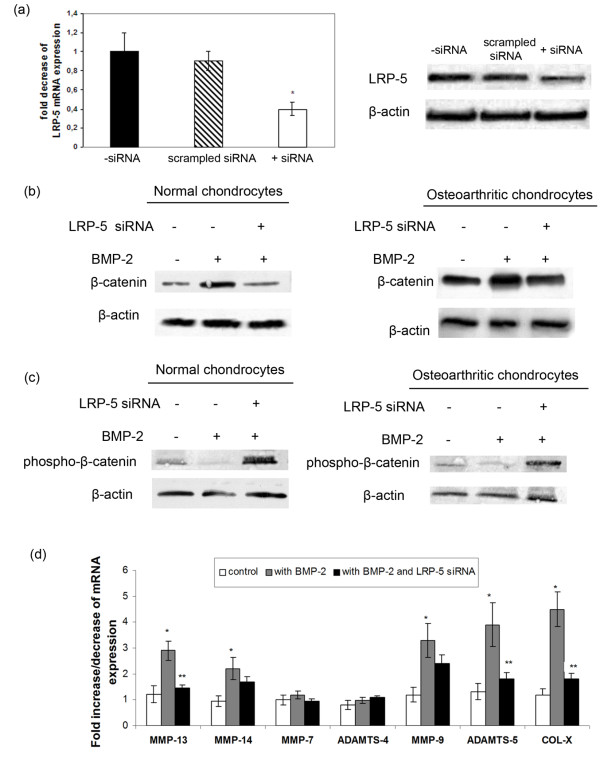
**Effect of BMP-2 treatment and LRP-5 silencing on β-catenin, matrix metalloproteinases (MMPs), and collagen X expression**. **(a) **The effects of siRNA against LRP5 on LRP5 mRNA and protein expression levels. (Error bars, SEM; *P < 0.05 siRNA-transfected cells versus untransfected. **(b) **Detection of β-catenin protein expression levels by Western blot analysis after LRP-5 siRNA transfection in BMP-2-treated normal and osteoarthritic chondrocytes. **(c) **Detection of phospho-β-catenin protein expression levels with Western blot analysis after LRP-5 siRNA transfection in BMP-2-treated normal and osteoarthritic chondrocytes. **(d) **Detection of MMPs, ADAMTSs, and collagen X expression levels with real-time PCR after LRP-5 siRNA transfection in BMP-2-treated normal and osteoarthritic chondrocytes. (Error bars, SEM; *P < 0.05 BMP-2-treated cells versus control. **P < 0.05 BMP-2 and LRP-5 siRNA-treated cells versus BMP-2-treated cells).

To provide evidence that BMP-2 stimulates catabolic enzymes and hypertrophic markers through Wnt/β-catenin signaling in osteoarthritic chondrocytes, we blocked LRP-5 mRNA expression by using siRNA against LRP-5 in BMP-2-treated normal and osteoarthritic chondrocytes and evaluated different MMPs (MMP-7, 9, 13, and 14), ADAMTSs (ADAMTS-4 and 5), and collagen X expression levels. siRNA against LRP-5 effectively inhibited LRP-5 expression, as we observed a downregulation of LRP-5 mRNA and protein expression in si-RNA-transfected chondrocytes compared with the untransfected and the scrambled siRNA-transfected chondrocytes 24 hours after siRNA transfection (*P *< 0.05; Figure 4a). Moreover, we found that LRP-5 silencing in BMP-2-treated normal and osteoarthritic chondrocytes resulted in downregulation of β-catenin protein levels (Figure 4b) and MMPs (MMP-9, 13, and 14), ADAMTS-5, and collagen X mRNA levels (Figure 4d). In addition, phospho-β-catenin protein levels were upregulated after LRP-5 siRNA transfection in BMP-2-treated normal and osteoarthritic chondrocytes (Figure 4c), suggesting that LRP-5 upregulation by BMP-2 contributed to Wnt/β-catenin signaling activation and chondrocyte catabolic activity and hypertrophy.

### Effect of activation of the Wnt/β-catenin signaling pathway by LiCl on the expression of genes implicated in catabolic action and hypertrophy of chondrocytes

**Figure 5:**
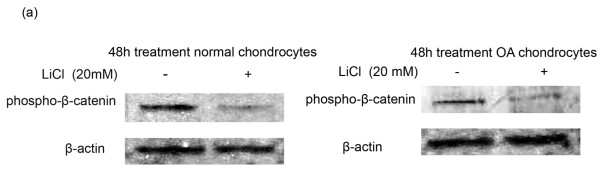
**Effect of LiCl treatment on phospho-β-catenin protein levels**. Phospho-β-catenin protein levels in normal and osteoarthritic chondrocytes after treatment with 20 m*M *LiCl for 48 hours. The results showed that phospho-β-catenin protein levels were significantly decreased in LiCl-treated normal and osteoarthritic chondrocytes compared with untreated. The results were normalized by using anti-β-actin polyclonal antibody.

**Figure 6:**
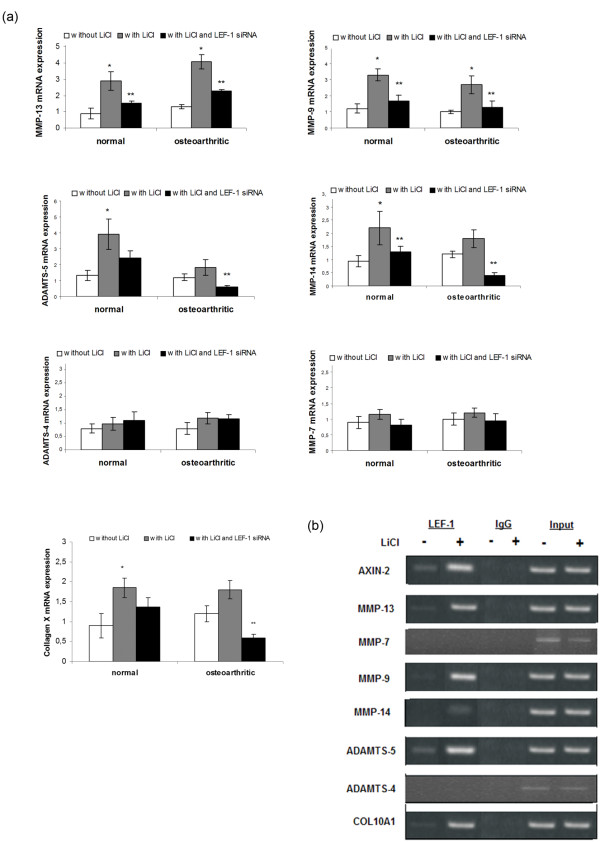
**Effect of LiCl treatment and LEF-1 silencing on matrix metalloproteinases (MMP)s, ADAMTSs, and collagen X expression**. **(a) **Detection of MMPs, ADAMTSs, and collagen X expression levels with real-time PCR after LEF-1 siRNA transfection in LiCl-treated normal and osteoarthritic chondrocytes. (Error bars, SEM; *P < 0.05 LiCl-treated cells versus control; **P < 0.05 LiCl and LEF-1 siRNA-treated cells versus LiCl-treated cells). **(d) **Occupancy of *MMPs, ADAMTSs*, and *COL10A1 *promoters in LiCl-treated and -untreated chondrocytes by LEF-1 with ChIP analysis. Input chromatin used as positive control, and IgG, as negative control.

To obtain direct evidence that the Wnt/β-catenin signaling pathway can trigger extracellular matrix degradation and hypertrophic chondrocyte differentiation in osteoarthritis, we activated the pathway *in vitro *by using LiCl in normal and osteoarthritic chondrocytes and evaluated the expression levels of basic catabolic (MMP-7, 9, 13, 14, ADAMTS-5, and 4) and hypertrophic markers (collagen X). We found that normal and osteoarthritic chondrocyte treatment with LiCl reduced phospho-β-catenin levels (Figure 5), suggesting the stabilization of β-catenin and the activation of Wnt canonic signaling. We then investigated the expression of different MMPs, ADAMTSs, and collagen X in treated and untreated-LiCl normal and osteoarthritic chondrocytes. We found that MMP-13 and MMP-9 mRNA levels were significantly upregulated after LiCl treatment in normal and osteoarthritic chondrocytes (*P *< 0.05), whereas MMP-14, ADAMTS-5, and collagen X expression was significantly upregulated in normal chondrocytes (*P *< 0.05) and showed a trend to increase in osteoarthritic chondrocytes (Figure 6a). siRNA against LEF-1 decreased MMP-13, 9, and 14 mRNA levels in LiCl-treated normal and osteoarthritic chondrocytes (*P *< 0.05), whereas ADAMTS-5 and collagen X mRNA expression was significantly downregulated in LiCl-treated osteoarthritic chondrocytes (*P *< 0.05) and showed a trend to decrease in LiCl-treated normal chondrocytes (Figure 6a). However, MMP-7 and ADAMTS-4 mRNA levels remained at the same levels after LiCl treatment and LEF-1 silencing (Figure 6a). To investigate whether LEF-1 binds to *MMP-9, 13, 14, ADAMTS-5*, and *COL10A1 *promoters through conserved LEF-1 binding sites and upregulates their expression, we performed a ChIP assay in chondrocytes after treatment with LiCl for 24 and 48 hours. ChIP assay revealed LEF-1 binding sites on *MMP-9 *(-737 to -733 from the ATG initiation codon), *MMP-13 *(from -1142 to -1138), *MMP-14 *(from -575 to -571), *ADAMTS-5 *(from -755 to -751), and *COL10A1 *(from -784 to -781) promoters, and the binding was stronger in treated-LiCl chondrocytes, as LiCl stabilizes β-catenin active form and increases nuclear β-catenin protein levels, enhancing thus the complex between β-catenin and LEF-1 and the subsequent binding on gene promoters, suggesting that these genes are Wnt targets in adult articular cartilage (Figure 6b). No LEF-1 binding sites were observed in *MMP-7 *and *ADAMTS-4 *promoter in the region from 1,500 base pairs upstream of the transcript start to 100 base pairs downstream of the coding-sequence start (Figure 6b). LEF-1 binding to *AXIN-2 *promoter served as positive control.

## Discussion

Articular chondrocyte proliferation, expression of hypertrophy markers, and remodeling of the cartilage matrix by proteases are among the main characteristics of osteoarthritis [1]. Recent studies have shown that events normally taking place in terminal chondrocyte differentiation in the growth plate are also observed during OA development, suggesting that signaling molecules, such as Wnts and BMPs, regulating chondrocytes activity in the growth plate may play a key role in osteoarthritis pathogenesis [7,8].

In the present study, we provide for the first time, to our knowledge, evidence for a cross-talk between BMP-2 and Wnt/β-catenin signaling pathways in osteoarthritic chondrocytes. Although BMPs are involved in all phases of chondrogenesis affecting chondrocyte differentiation and cartilage anabolism [28-30], recent studies have shown that BMPs can also have harmful effects on articular cartilage [31]. However, their role in osteoarthritis is not completely elucidated. We evaluated BMP-2, 4, and 7, as well as their receptors, BMPR-IA and BMPR-IA expression levels in osteoarthritic and normal chondrocytes and found that osteoarthritic chondrocytes exhibited significantly higher BMP-2, 4, and BMPR-IA mRNA and protein levels, suggesting the involvement of BMP signaling in osteoarthritis progression. Previous studies have shown that BMP-2 can be activated by IL-1 and TNF-α in normal and osteoarthritic chondrocytes [36] and that it can stimulate the synthesis of matrix molecules and MMPs expression by modulation of chondrocyte differentiation [37,38]. In addition, mice deficient in type 1 receptors Bmpr1a or Bmpr1b in cartilage develop severe generalized chondrodysplasia, demonstrating that BMP signaling is required for chondrocyte proliferation, survival, and differentiation [39].

It has been suggested that BMP-2 modulates β-catenin signaling through stimulation of Wnts, LRPs, and Frizzled receptors expression in mesenchymal cells and osteoblasts [34,40]. To investigate the role of BMP-2 on the Wnt/β-catenin signaling-pathway activation in osteoarthritic chondrocytes, we evaluated β-catenin and LRP-5 levels after treatment of normal and osteoarthritic chondrocytes with BMP-2. We observed that BMP-2 enhances the nuclear-active form of β-catenin protein levels, decreases phospho-β-catenin protein levels, and increases LRP-5 mRNA and protein levels, which were found significantly upregulated in osteoarthritic chondrocytes compared with normal. In addition, blocking LRP-5 expression in osteoarthritic chondrocytes resulted in a significant decrease in MMP-13 expression, the basic catabolic enzyme of osteoarthritis [21]. All of these findings suggest LRP-5 involvement in the increased activation of Wnt/β-catenin signaling and cartilage degradation in osteoarthritis. No difference was observed in the nuclear-active form of β-catenin levels in BMP-4-treated chondrocytes, providing evidence on the Wnt/β-catenin signaling-pathway activation by BMP-2 and not by BMP-4.

To investigate further the molecular mechanism involved in the BMP-2-induced LRP-5 upregulation, we examined the effect of BMP-2 through Smads binding on *LRP-5 *promoter activity in chondrocytes. Chromatin immunoprecipitation indicated that BMP-2 directly modulates LRP-5 expression, as we found, for the first time, that Smads complexes bind to SBEs on the *LRP-5 *promoter, suggesting the BMP-2 induced-Wnt/β-catenin signaling-pathway activation through direct modulation of LRP-5 expression. Previous studies have shown that osteoblast differentiation and new-bone formation require the interaction of BMP-2 and the Wnt/β-catenin signaling pathway [41,42]. BMP-2-enhanced LRP-5 expression has a strong catabolic activity in chondrocytes, as LRP-5 silencing inhibited BMP-2-induced-β-catenin protein levels, MMPs, and collagen X expression, whereas increased phospho-β-catenin protein levels, providing evidence on the involvement of BMP-2-modulated β-catenin signaling in OA progression.

The Wnt/β-catenin signaling pathway participates in normal adult bone and cartilage biology and seems to be involved in cartilage degeneration and subsequent OA progression [43,44]. To determine whether changes in the Wnt-signaling pathway are associated with osteoarthritis, we evaluated the expression levels of Wnt transcription factors, LEF-1 and TCF-4, and phospho-β-catenin in osteoarthritic and normal articular chondrocytes. We observed that LEF-1 mRNA and protein-expression levels were significantly increased in osteoarthritic chondrocytes, whereas phospho-β-catenin protein levels were significantly decreased in osteoarthritic chondrocytes, suggesting the excessive activation of canonic the Wnt-signaling pathway in osteoarthritis.

To test for a possible association between the LEF-1 transcription factor of the Wnt/β-catenin signaling pathway and catabolic action, as well as hypertrophy in osteoarthritic chondrocytes, we activated Wnt/β-catenin signaling by using LiCl in cultured chondrocytes. LiCl is often used to mimic canonic Wnt signaling, as it inhibits GSK-3β, therefore stimulating downstream components of the Wnt-signaling pathway in an LRP-5-independent manner [45]. The activation of the canonic Wnt-β-catenin signaling by LiCl is not modulated by LRP-5 phosphorylation, and until now, differences in phospho-LRP-5 protein levels between OA and normal cartilage have not been reported. We observed that experimental activation of Wnt/β-catenin signaling induced significant upregulation of catabolic enzymes such as MMP-9, 13, 14, aggregenases, as ADAMTS-5 and hypertrophic marker, collagen X. The upregulation of the above genes takes place in a direct manner, as we demonstrated, conserved LEF-binding sites in *MMP-9, 13, 14, ADAMTS-5*, and *COL10A1 *promoters, responsible for their promoter activity and is associated directly with the β-catenin/LEF-1 complex. Moreover, LEF-1 downregulation using siRNA reduced MMPs, ADAMTS-5, and collagen X mRNA expression, whose levels increased after treatment with LiCl, providing strong evidence of gene-expression regulation of catabolic factors by LEF-1. No upregulation was observed in MMP-7 and ADAMTS-4 levels, as no conserved LEF-binding sites were found on their promoters. It has been shown that Lef-1 binding to the 3' region of *mmp-13 *is involved in the transcriptional regulation of the *mmp-13 *gene in mouse chondrocytes [46]. It can be suggested that the Wnt/β-catenin signaling pathway promotes chondrocytes catabolic activity, degrading not only collagen II, as evidenced by the observed increased expression of MMP-13 and 14, but also other extracellular matrix molecules, such as fibronectin and aggrecan, through enhancement of MMP-9 and ADAMTS-5 expression, respectively. It has been shown that MMP-9, a gelatinase with broad substrate specificity, contributes to fibronectin degradation and increases the fibronectin fragments, which have been shown to be elevated in OA [47,48], whereas ADAMTS-5, one of the most efficient aggrecanases, degrades aggrecan, the major proteoglycan in cartilage [49]. In additional studies in a murine model of osteoarthritis, deletion of ADAMTS-5 provided significant protection against proteoglycan degradation *ex vivo *and decreased the severity of osteoarthritis [50]. We also found that the Wnt/β-catenin signaling pathway contributes to the hypertrophic differentiation of chondrocytes, increasing collagen X expression, the basic hypertrophic marker of chondrocytes. We therefore showed that the Wnt/β-catenin signaling pathway plays a major role in OA progression, as articular chondrocytes, after experimental activation of the Wnt/β-catenin signaling pathway, cannot maintain the characteristics of the permanent cartilage but instead enhance extracellular matrix degradation, evidenced by increased MMPs and ADAMTS-5 expression, and mature to hypertrophic through stimulation of collagen X expression.

## Conclusion

In conclusion, we demonstrated, for the first time to our knowledge, that the BMP-2-induced Wnt/β-catenin signaling pathway activation through LRP-5 induces chondrocyte catabolic action and hypertrophy, providing thus novel and direct evidence on the role of BMP-2 mediated by Wnt/β-catenin signaling in osteoarthritis progression.

## Abbreviations

BMPs: bone morphogenetic proteins; BMPR-IA: bone morphogenetic protein receptor-IA; BSA: bovine serum albumin; ChΙP: chromatin immunoprecipitation; COL10A1: collagen X; Dkk-1: Dickkopf-related protein 1; DMEM/F-12: Dulbecco Modified Eagles Medium/Ham F-12; FBS: fetal bovine serum; FRZB: Frizzled-related protein; GAPDH: glyceraldehyde 3-phosphate dehydrogenase; LEF-1: lymphoid enhancer factor-1; LRP-5: low-density-lipoprotein receptor-related protein 5; MMPs: matrix metalloproteinases; OA: osteoarthritis; SBEs: Smad-binding elements.

## Competing interests

The authors declare that they have no competing interests.

## Authors' contributions

IP performed the experiments and drafted the manuscript, KNM provided cartilage samples, clinical evaluation of patients, and helped in data interpretation, and AT conducted data analysis, results interpretation, and finalized the manuscript. All authors read and approved the final manuscript.
